# An insider's view of the new diagnostic and statistical manual of North American psychiatry (DSM-5)

**Published:** 2013-05-30

**Authors:** Javier I Escobar

**Affiliations:** Associate Dean for Global Health, Robert Wood Johnson Medical School, New Brunswick, New Jersey, USA escobaja@umdnj.edu

## Introduction

The relevance/ visibility of psychiatric disorders within the realm of medicine has significantly increased in recent times, possibly due to their high frequency and the negative impact they have on cost, disability and quality of life. The subjective nature of these disorders, their clinical complexity and the absence of reliable markers, keep us completely dependent on anamnesis and clinical examination. All of this, forces us, periodically, to review and refine diagnostic systems, hoping to improve the recognition and effective management of mental disorders. The scientific progress in basic neuroscience, observed during and following the "decade of the brain", coupled with a lack of satisfaction with the existing system (DSM-IV) suggested it was the moment to embark in this task, hence the process leading to DSM-5.

## The DSM System:

The official classification of psychiatric disorders in North America, a process coordinated by the committee on nomenclature and statistics of the American Psychiatric Association (APA), began with the publication of the Diagnostic and Statistical Manual (DSM), first published in 1952. DSM-I, with 20 editions that were widely distributed in the US and abroad, was the official system for 15 years, until a new committee, prepared DSM-II that became official in 1968, following the publication of the eight edition of the international classification of diseases (ICD-8). These first two versions of the manual (DSM-I and II) were descriptive systems, without specific criteria, open to interpretation and filled with theoretical influences, since they appeared during the hegemony of the psychoanalytic approach of the first half of the 20th century in North American psychiatry. Following the decline of psychoanalysis, the emergence of biological psychiatry and the "psychopharmacological revolution," two pioneers, Eli Robins and Samuel Guze, armed with outstanding clinical skills, astute minds and Anglo-Saxon philosophical positivism, started describing and cataloguing clinical syndromes at their headquarters in Washington University and Barnes Hospital in St Luis. This group created the "St Louis Criteria" whose publication in the 'Archives of General Psychiatry' in 1972 immortalized a "lucky" young resident, John P Feighner, who convinced his parsimonious professors, to put in writing the list of criteria resulting from detailed clinical descriptions, and buttressed by careful follow up studies and successive validation steps^1^ . The "Feighner criteria", as they became popularly known, provided diagnostic criteria for 14 mental disorders, the only ones they considered valid at that time. This began the modern era of psychiatric nosology in North America. Robert Spitzer, a psychiatrist from New York City, influenced by the "spirit of St Louis", developed the research diagnostic criteria (RDC) by the end of the 1970s, which included Eli Robins as a coauthor. Spitzer would then become head of the committee on nomenclature and statistics of the APA that developed the operational criteria included in DSM-III (1980), a paradigm change, providing detailed lists of criteria for 265 diagnoses in a "Chinese menu" format for making specific choices. DSM-III started placing particular emphasis on diagnostic reliability or agreement among observers more than on validity. The next revision of the DSM system, DSM-III-R, a process also led by Spitzer. DSM-III-R refined criteria for several diagnoses and added 27 new entities thus totaling 292 different categories. DSM-IV then followed in 1994, and while Spitzer remained involved, the process was led by another New Yorker, Alan Frances. DSM-IV and DSM-IV-TR, the edition that followed, added other 69 diagnoses for a grand total of 361 separate categories, a significant "inflation" of the 14 diagnoses originally proposed by the St Louis group!^2^

## The DSM-5 Process:

The evolution towards DSM-5 began more than a decade ago, stimulated by the need for a change in paradigm and the belief that DSM III and IV criteria do not describe or draw natural phenomena precisely ("did not carve nature by the joints"), forcing practitioners, particularly the most scrupulous ones, to use "nonsensical" categories such as diagnoses "NOS" (not otherwise specified). Thus, depressive disorder NOS would become the most commonly made diagnosis in the United States. Moreover, as DSM-III and IV criteria resulted from a strong desire to increase reliability (agreement across evaluators), they sacrificed validity, treating each disorder as a categorical entity, discontinuous from normality and from other disorders. This tendency to split syndromes into smaller parts, led to the creation and reification of trivial entities that after being included in the "psychiatric bible" (as DSM criteria is popularly called) took a life of their own. Researchers in the neurosciences have been arguing for years, that when criteria such as DSM-IV are rigidly applied, this limits progress in research and therapeutic developments, and the task of identifying genetic influences for these disorders becomes quite arduous^3^. It is not surprising therefore, to observe that very different disorders such as autism, depression, bipolar or psychotic disorders, appear to share common genes, and conversely, that a single category such as schizophrenia, is linked to multiple, unrelated genes.

Thus, at the beginning of the DSM-5 process, we thought that this could be the moment to introduce a new classification that incorporated a dimensional approach, consider advances in neuroscience such as brain circuitry and introduce biological markers in the diagnostic process.

Over a decade ago, a series of international conferences with specific focus on key topics (e.g., dimensional diagnosis, personality disorders, addictions, dementias and several others), proposed a number of new initiatives in each area, thus setting the pace for the DSM-5 process.

In August 2007, the APA formally announced the creation of the DSM-5 task force in which I had the honor of being included, together with 23 other colleagues, a majority of them leading academic psychiatrists in the country. Initial discussions led to the creation of 16 workgroups, each led by a member of the task force, and each focusing on specific sets of disorders (e.g., addictions, personality disorders, anxiety disorders, mood disorders, somatic symptom disorders, etc.). These workgroups, started adding consultants and collaborators, and eventually accumulated more than 400 individuals that participated in this process in the past 2-3 years.

On the basis of thorough literature reviews, expert opinions and active debates in efforts to reach consensus, each work group made specific proposals to change, modify, or eliminate DSM-IV criteria and drafted new diagnoses for DSM-5. However, these proposals did not go directly to the task force, but had to undergo first reviews by a Scientific Advisory Committee (SCR), independent from the task force, that evaluated scientific aspects of the proposals as well as a Community and Public Health Committee (CPHC) that assessed practical issues and public health relevance of changes and new diagnoses. These two advisory groups graded changes and new diagnoses and recommended approval or disapproval. These recommendations then went to the task force, and we discussed each proposal individually and voted to either approve or disapprove it, passing our recommendations to the APA Board of Directors for final approval.

What is new in DSM-5? DSM-5 will be introduced on May 18th at the meeting of the APA in San Francisco. It is not surprising to observe, that the initial enthusiasm regarding innovations and revolutionary (paradigmatic) changes (jocularly described by one of our colleagues as "Robins and Guze on steroids"), was eventually tempered by realities and practical issues such as the additional burden that adding hundreds of new dimensional codes would bring to the busy practicing psychiatrist and the attachment of experts and associations to certain diagnostic criteria and labels, that created significant resistance to change. Thus, at the end, there were only modest changes and "tweaking" of the criteria, which I will try to summarize below.

In brief, major changes in DSM-5 relative to DSM-IV include:

1-The DSM-5 manual is made up of 3 parts or sections. Section I includes the introduction to the manual and the list of diagnostic criteria grouped according to common elements. Section II is the main section of the manual. It includes all the new diagnoses that made the cut and those changes in DSM-IV criteria that received formal approval, as well as the diagnoses from DSM-IV that required no change. Section III, includes new diagnoses and changes, that while promising, are not yet "ready for prime time" and for which, there is a need for additional research. New disorders included in section II, include "hoarding disorder", "somatic symptom disorders" (replacing somatoform disorders), "autism spectrum disorder" (replacing autistic disorder and Asperger disorder), as well as "nightmare disorder", "insomnia disorder", "suicide disorder" and several others.

Disorders included in section III include "alternate personality disorders" (Section II retained all DSM-IV personality disorders), dimensional or cross-cutting measures, attenuated psychosis syndrome, internet addiction disorder, internet gaming disorder, caffeine use disorder, olfactory reference disorder, protracted bereavement disorder and several others.

2-DSM-5 eliminated the multiaxial system from DSM-III and IV and will be using only a single Axis, consistent with ICD criteria.

3-A number of DSM-IV diagnoses were eliminated from DSM-5 (subtypes of schizophrenia, several childhood diagnoses, substance dependence disorders, and several others).

4-Criteria for several disorders were modified in order to make them more precise. For example, addiction disorders (combining abuse and dependence), post traumatic stress disorder, attention deficit hyperactivity disorder, major depressive disorder, bipolar disorder and several other diagnoses underwent some revisions.

5-Grading of severity (mild, moderate, severe) is included for several disorders.

6- The text was expanded to describe cultural and gender elements that are relevant for each disorder.

## Controversies:

During the past 2-3 years, once diagnostic proposals were published and distributed for comments, there has been intense debate, frequently reaching the leading news media, reflecting the importance and visibility of the DSM in the United States.

Curiously, Alan Frances, the person who led DSM-IV, has become the "nemesis" of DSM-5, from his retirement villa in sunny San Diego, California. Frances has been leading and coordinating a broad campaign to discredit DSM-5 utilizing such venues as "Psychology Today", "Psychiatric Times", "the Huffington Post" and also groups and "lobbies" opposing psychiatric diagnoses to denigrate the new system. The impression is that Frances and others (including Spitzer) resented their exclusion from the DSM-5 process by APA governance, but their reaction appears quite excessive.

## NIMH, the APA and DSM-5:

The director of NIMH, Thomas Insel, in collaboration with Bruce Cuthbert, a psychologist from the institute, proposed a couple of years back, a rather esoteric system called Research Domain Criteria o R-DoC^4^. While some viewed this as an alternative to DSM-5, RDoC appeared to be simply a proposal to approach mental phenomena biologically, based on "molecules", "circuits", and "positive or negative valences", more than a classification system per se^5^. However, a couple of weeks before the release of DSM-5, Insel announced in his internet blog his rejection of the DSM system and his intent to have the institute exclude DSM criteria from grant applications, forcing research grant applicants to use his RDoC instead^6^.

Obviously, as discussed at the outset of the DSM-5 process, we hoped that as neuroscience research increases knowledge and provides "hard", consistent evidence on brain function, brain circuits and reliable markers, that this might lead to a more precise classification of at least some heterogeneous disorders such as schizophrenia. Unfortunately, as eloquently expressed by David Kupfer, the chair of the DSM-5 task force, reliable diagnostic markers remain "disappointingly distant". Personally, I view Insel's attitude and comments as being quite inappropriate, coming from the director of one of the leading NIH institutes. This attitude is not only deeply reductionist with an intense biological bias, but it ignores the complexity of mental disorders, distancing him from clinical realities and the practice and provision of psychiatric services. It also creates a rather unique conflict of interest, tying funding decisions to the use of a diagnostic strategy he devised himself. Despite the announcement in the media of a "battle between APA and NIMH", the response of APA leadership was initially cautious, not challenging, while curiously, practicing psychiatrists thought that neither DSM-5 or RDoC will make a difference whatsoever for their lives^7^.

Two days ago (May 13th), a joint release from Insel and Jeffrey Lieberman, the President elect of APA, sets a more friendly tone, acknowledging that "DSM-5 and ICD-10 represent the best information currently available and remain the contemporary consensus standard of how mental disorders are diagnosed and treated", while emphasizing the need for searching more reliable markers and making it clear that "DSM-5 and RDoC represent complementary, not competing frameworks"^8^.

So, I am glad the "fuss" has dissipated, and in a couple of days, I will be heading west, to San Francisco, to "touch and smell" for the first time the complete DSM-5 manual, hoping not to find many "surprises" (and hopefully, only pleasant ones). From a practical perspective, I remain convinced that DSM-5 will be successfully implemented, tested and improved as new knowledge accrues, so that we continue to view it as a "living" document, a draft in need of constant improvement, not the "psychiatric bible".
